# Knowledge, Attitude, Awareness, and Perceptions among Physicians toward Antibiotic Resistance in Hospitals in South Palestine

**DOI:** 10.1055/s-0043-1764374

**Published:** 2023-03-24

**Authors:** Hussein Jabbarin, Inad Nawajah, Hatem A. Hejaz

**Affiliations:** 1 College of Nursing, Hebron University, Hebron, Palestine; 2 College of Science & Technology, Hebron University, Hebron, Palestine; 3 Faculty of Pharmacy, Arab American University, Jenin, Palestine

**Keywords:** antibiotic resistance, physicians, knowledge, attitude, awareness, perceptions, antibiotics

## Abstract

**Background** Antibiotic resistance is a global problem, and the World Health Organization has made this problem one of its priorities for solving. Therefore, a survey was carried out to investigate the knowledge, attitude, awareness, and perceptions of antibiotic resistance among physicians and to assess the correlation between the knowledge of antibiotic resistance and their years of experience in some Palestinian hospitals.

**Methods** This was a cross-sectional study that targeted physicians who are working in different healthcare facilities in Hebron and Bethlehem governorates. We used a questionnaire for data collection. The questionnaire consists of 42 questions to measure the knowledge, attitudes, awareness, and perceptions of antibiotic use and resistance.

**Results** The response rate was 91.33% (137 of 150 physicians completed the questionnaire). The participants' ages ranged from 25 to 56 years, and the majority were males ( *n* = 116, 84.7%) working in governmental hospitals ( *n* = 83, 60.6%). Of physicians, 69.3% ( *n* = 95) perceived antibiotic resistance as a very important worldwide problem, while 54.7% ( *n* = 75) perceived a very important problem in the country, 54.0% ( *n* = 74) a very important problem in their hospital, and 59.1% ( *n* = 81) a very important problem in their departments. Methicillin-resistant Staphylococcus aureus was the most known antibiotic-resistant bacteria followed by extended-spectrum beta-lactamases. Only 47 physicians (34.3%) think that antibiotics are not used appropriately in their department. Respondents' physicians showed that the development of antibiotic resistance was due to various factors that include self-medication n= (92, 67.2%), overuse of antibiotics ( *n* = 83, 60.6%), and uncompleted treatment (n= 87, 63.5). Senior specialists/consultants were found to be more knowledgeable about antibiotic resistance.

**Conclusion** In our survey, physicians showed variable knowledge and perceptions of antibiotic resistance. Introducing educational programs is necessary to improve their understanding and perceptions of antibiotic resistance, as well as their attitude toward antibiotic use.

## Introduction

According to the World Health Organization (WHO), antibiotics are the most frequently prescribed drugs worldwide. 1 2 Even though antibiotics have a great advantage in reducing the incidence and spread of infectious diseases worldwide, they are frequently misused, leading to the spread of multiple resistant bacterial strains. A growing number of infections—such as pneumonia, tuberculosis, gonorrhea, and salmonellosis—are becoming harder to treat as the antibiotics used to treat them become less effective. 3 The WHO defines antimicrobial resistance as "the change in a microorganism that causes it to become resistant to an antimicrobial drug that was previously effective against it." 4 As a result, the antibiotics became ineffective and infections persist in the body, increasing the risk of spreading to other people, and leading to longer hospital stays higher medical costs, and increased mortality. Antibiotic resistance is one of the biggest threats to global health. It can affect anyone, of any age, in any country. Each year in the United States, at least 2 million people get an antibiotic-resistant infection, and at least 23,000 people die. Antimicrobial resistance (AMR) is one of the major health issues worldwide. Clinicians should play a central role to fight AMR, and medical training is a pivotal issue to combat it; therefore, assessing levels of knowledge, attitudes, and practices among young doctors is essential for future antimicrobial stewardship programs. Repeated and improper use of antibiotics is the main reason for the bacteria's resistance to the drugs. 5

According to the WHO, more than 50% of medicines are incongruously prescribed, dispensed, and utilized. Furthermore, around half of the patients take their medicines incorrectly. 6 The irrational use of medicine is an alarming situation in developing countries due to their weak healthcare systems where the monitoring mechanism for routine medicine use is often not properly developed. 7 Physicians usually prescribe antibiotics without scientific bases when they are concerned about complications of infections, or when desiring to achieve patients' expectations. These nonscientific prescriptions have a great role in the development of antibiotic resistance. In addition, antibiotics in many developing countries including Palestine are sold over the counter without prescription; this is also an important reason for the increase in bacteria resistance against the antibiotics. Multiple studies have been conducted to assess knowledge, attitude, and perceptions (KAP) among physicians toward antibiotic resistance. Many of those studies have been carried out in the United States, Turkey, Pakistan, India, Malaysia, Palestine, France, Scotland, and other countries. 8 9 10 11 12 13 14 Understanding the knowledge, attitudes, and practices of physicians toward antibiotic resistance is the key to developing interventions aimed at behavior change. The survey aimed to investigate physicians' KAP of antibiotic resistance in southern Palestinian hospitals. To the best of our knowledge, this study is the first in Palestine about antibiotics resistance among physicians in the south of Palestine and the second study about antibiotics resistance among physicians in the country as the first study was performed by Abu Taha et al in 2019 about antimicrobial resistance and prescribing among physicians in governmental hospitals in North Palestine. 13 Our study is more comprehensive and covered the other part of Palestine and included private and governmental hospitals in South Palestine, while Abu Taha et al 13 assessed the KAP about antimicrobial resistance and prescribing among Physicians in governmental hospitals in North Palestine.

## Materials and Methods

### Study Design and Settings

We conducted a cross-sectional study among physicians in the southern part of the West Bank (Hebron and Bethlehem governorates) in March 2021; the study was conducted for two weeks. We included governmental and nongovernmental/private hospitals. Among the selected hospitals, four were governmental (Alia Governmental Hospital, Mohammad Ali Al Muhtaseb Hospital, Yatta Governmental Hospital, and Beit-Jala Hospital) and the other four were nongovernmental/private (Al-Ahli Hospital, Al-Mezan Hospital, Palestinian Red Crescent Hospital, and Holy Family Hospital). These hospitals are located in different cities in the two governorates.

### Study Population

A self-administered questionnaire was distributed in the hospitals among physicians of all levels who prescribe antibiotics including general physicians, medical officers/ residents (medical graduates engaged in specialized practice under supervision), senior residents/ specialists, senior specialists/ consultants, and also the physicians working at departments of surgery, internal medicine, pediatrics, emergency, intensive care units, obstetrics, and gynecology. One-hundred thirty-seven of 150 physicians in total were found and considered eligible to participate in our study (response rate: 0.91). Physicians who have a medical degree and doing internship training but do not have a license to practice medicine were excluded from the study because they do not often prescribe antibiotics during their training (13 physicians were excluded from the study).

### Survey Instrument

We used the questionnaire developed for assessing antimicrobial use in the Korle-Bu Teaching Hospital. 15 The adapted questionnaire is a 40 items questionnaire; we added only two questions; one is about attending workshops/ courses related to antibiotic resistance, and the other one is about the department in the hospital the participants visited. Thus, the distributed questionnaire became of 42 questions to measure the KAP of antibiotics use and resistance. Also, before starting the study, our questionnaire was translated into the Arabic language and reviewed by four expert physicians on infectious diseases to assess the relevance and wording of the questions as well as the accuracy of the translation into Arabic (i.e., the Arabic version was distributed to the physicians).

The questionnaire is divided into four parts, the first included demographics and general information data (including age, gender, level of training, duration of practice since completion of medical school, hospital, department of practice, and the number of hours of antibiotic resistance-related workshops courses attended). The second part included questions assessing the perceptions on the importance of antibiotic resistance as a problem at the global, national, hospital, and department levels. It used 4-point Likert-scale response options (1 = strongly agree, 2 = agree, 3 = disagree, and 4 = strongly disagree). The questions using the 4-point Likert scale were dichotomized such that correct answers for KAP and preparedness were noted if a participant agreed or strongly agreed (scale: 1–2, high mean) in a positive question or disagreed or strongly disagreed (scale: 3–4) in a negative question.

The third part of the questionnaire acquired information about perceptions on causes of antibiotic resistance, which are (overuse of antibiotics, ineffective selection of antibiotics, self-medications, and low dosage), and to what extent each contributes to the development of antibiotic resistance. The fourth and last part included questions on knowledge and attitude on antibiotic-resistant bacteria of public health importance (including methicillin-resistant Staphylococcus aureus [MRSA], extended spectrum beta-lactamase-producing enterobacteria [ESBL], carbapenem-resistant enterobacteria [CRE], and vancomycin-resistant enterococci [VRE]). To assess knowledge of the prevalence of antibiotic-resistant bacteria of public health importance, the last question asked the physicians to choose the correct order of prevalence for MRSA, ESBL, CRE, and VRE.

### Survey Administration

Printed questionnaires (Arabic version) were distributed among physicians for 2 weeks in March 2021. Each questionnaire included a cover letter explaining the purpose of the study and assuring participants that the privacy and confidentially of collected data will be maintained.

### Statistical Analysis

The entire study data were entered in Microsoft Excel and analyzed using the Statistical Package for Social Sciences Version SPSS 26 (IBM, United States). Frequency and percentages among descriptive statistics were used to describe the data. Proportions were calculated for categorical variables; mean and standard deviations were calculated for continuous variables. Descriptive statistics provide summary information about data, for example, the number of respondents who are male or female. Here we collected this information from 137 respondents out of 150 physicians (response rate of 0.91).

The KAP of antibiotic resistance among physicians scores according to demographic characteristics were compared with independent- samples t-test and one-way analysis of variance as appropriate. Finally, the Pearson correlation (R) test was used to test the relation between the total score of knowledge and mean scores of antibiotics attitude and practice. The strength of the correlation between knowledge, attitude, and practices of antibiotics is described as follows: 0.0–0.19 “very weak,” 0.20–0.39 “weak,” 0.40–0.59 “moderate,” 0.60–0.79 “strong,” and 0.80–1.0 “very strong.” In all cases, the *p* -value less than 0.05 were taken as statistically significant.

## Results

### Demographics

About 91% of four different specializations have completed the survey as 137 of 150 physicians filled out the questionnaire. Their age ranged from 25 to 56 years and 116 participants were males and 21 females. The mean age of the respondents was 32.40 ± 3.15 years. Four different specializations of the physicians participated in the study; 22 (16.1%) general physicians, 54 (39.4%) medical officers/residents, 43 (31.4%) senior residents/specialists, and 18 (13.1%) senior specialists/consultants. Moreover, the average years of practice among the study participants were 6.67 ± 2.35 years. Fifty of them (36.5%) attained continuing education/workshops related to antibiotic resistance in the last 3 years, with an average of 14.6 hours per year ( Table 1 ).

**Table 1:** Sociodemographic characteristics of the study participants

		Frequency	Percentage (%)
Gender	Male	116	84.7
	Female	21	15.3
Level of training	General physician	22	16.1
	Medical officer/resident	54	39.4
	Senior resident/specialist	43	31.4
	Senior specialist/consultant	18	13.1
Hospital	Governmental	83	60.6
	Nongovernmental (private)	54	39.4
Attending workshops/ courses related to antibiotic resistance (continuing education)	Yes	50	36.5
	No	87	63.5
Age of participants in years (mean ± SD)	32.40 ± 3.15		
Years of practice among study participants. (mean ± SD)	6.67 ± 2.35		
Hours of continuing education among study participants (mean ± SD)	14.66 ± 17.54		

Abbreviation: SD, standard deviation.

Table 2 shows the hospital department that is the most visited by participant physicians; the pediatric department is the most visited.

**Table 2:** Most visited hospital department

Department	Number	Percentage (%)	Department type
Coronary care unit (CCU)	11	8.0	Inpatient wards
Emergency department (ED)	13	9.5	Inpatient wards
Intensive care unit (ICU)	3	2.2	Inpatient wards
Neurology	2	1.5	Inpatient wards
Neonatal intensive care unit (NICU)	2	1.5	Inpatient wards
**Total**	**31**	**22.6%**	**Inpatient wards**
Ear, nose, and throat (ENT)	1	0.7	Outpatient
Gynecology	25	18.2	Outpatient
Medicine	12	8.8	Outpatient
Nephrology	2	1.5	Outpatient
Oncology	2	1.5	Outpatient
Orthopaedics	14	10.2	Outpatient
**Pediatric**	**26**	**19.0**	**Outpatient**
Surgical	19	13.9	Outpatient
Urology	5	3.6	Outpatient
**Total**	**106**	**77.4%**	**Outpatient**

### The Perceived Problem of Antibiotic Resistance

Of physicians' respondents, 95 (69.3%) perceived antibiotic resistance as a very important worldwide problem, 75(54.7%) as a very important problem in the country, 74(54.0%) as a very important problem in their hospital, and 81(59.1%) a very important problem in their departments ( Table 3 ). The knowledge about antibiotic-resistant of the four bacteria groups presented to respondents (VRE, CRE, ESBL, and MRSA) is shown in Table 3 . The participants have the highest knowledge of MRSA (94.9%, *n* = 130/137) and the lowest knowledge of CRE (73.0%, *n* = 100/137).

**Table 3:** Physicians' perceptions and knowledge of antibiotic resistance

Physicians' perception and knowledge	Number of the respondent (%)			
Perceptions	Very important	Important	Not important	Don't know
Grade the level of antibiotic resistance worldwide	95 (69.3)	39 (28.5)	2 (1.5)	1 (0.7)
Grade the level of antibiotic resistance in the country	75 (54.7)	51 (37.2)	7 (5.1)	4 (2.9)
Grade the level of antibiotic resistance in the hospital	74 (54.0)	56 (40.9)	3 (2.2)	4 (2.9)
Rate the impact of antibiotic resistance on patient safety in your department	81 (59.1)	51 (37.2)	3 (2.2)	2 (1.5)
Do you think antibiotics are used appropriately in your department?	**Yes**		**No**	
	90(65.7)		47(34.3)	
Knowledge	**VRE**	**CRE**	**ESBL**	**MRSA**
Do you know about the following resistant bacteria?	114 (83.2)	100 (73.0)	117 (85.4)	130 (94.9)
Have you ever managed a patient with infections by the following resistance bacteria?	67 (48.9)	65 (47.4)	96 (70.1)	105 (76.6)
Do you think patients in this hospital are at risk of the following resistance bacteria?	93 (67.9)	77 (56.2)	100 (73.0)	106 (77.4)
Do you think the hospital has a problem with the following resistant bacteria?	78 (56.9)	76 (55.5)	90 (65.7)	100 (73.0)
**What is the extent of the hospital's problem with the following resistant bacteria?**				
Very serious	20 (14.6)	21 (15.3)	35 (25.5)	54 (39.4)
Serious	52 (38.0)	52 (38.0)	48 (35.0)	43 (31.4)
Not serious	6 (4.4)	3 (2.2)	7 (5.1)	3 (2.2)
Indicate the correct order of prevalence for the following resistant bacteria in the hospitals Frequency of respondents (%)				
MRSA > ESBLs> CRE > VRE	ESBLs > MRSA> VRE > CRE	ESBLs > MRSA > CRE <VRE		Don't know
60(43.8)	29(21.2)	24(17.5)		23(16.8)

Abbreviations: CRE, carbapenem-resistant enterobacteria; ESBL, extended spectrum beta-lactamase; MRSA, methicillin-resistant Staphylococcus aureus; VRE, vancomycin-resistant enterococci.

### Perceptions on Causes of Antibiotic Resistance

Different factors were significantly perceived by the majority of physicians as very important causes of antibiotic resistance ( Table 4 ) that is the overuse of antibiotics in hospitals 65(47.4%), overuse of antibiotics in the population 83 (60.6%), ineffective antibiotic control in hospital 60 (43.8%), and self-medication 92 (67.2%). Less than half of the physicians, 46, (33.6%) considered poor quality antibiotics and too low antibiotic dosages 47 (34.3%) as very important causes of antibiotic resistance. Thus, five factors were identified as being the most important causes of antibiotic resistance, that is, self-medication, uncompleted antibiotic therapy, overuse of antibiotics in the population overuse of antibiotics in hospitals, and ineffective antibiotic control in hospitals.

**Table 4:** Perceptions of causes of antibiotic resistance

Respondents' perceptions of causes	Number of the respondent (%)			
To what extent do the factors below contribute to the development of antibiotic resistance	Very important	Important	Not important	Don't know
Overuse of antibiotics in the hospitals	65 (47.4)	65 (47.4)	6 (4.4)	1 (0.7)
Overuse of antibiotics in the population	83 (60.6)	47 (34.3)	6 (4.4)	1 (0.7)
Ineffective antibiotic control in hospitals	60 (43.8)	65 (47.4)	8 (5.8)	4 (2.9)
Use of antibiotics in animals	19 (13.9)	33 (24.1)	24 (17.5)	61 (44.5)
Poor-quality antibiotics	46 (33.6)	66 (48.2)	17 (12.4)	8 (5.8)
Too low antibiotic dosages	47 (34.3)	71 (51.8)	16 (11.7)	3 (2.2)
Self-medication	92 (67.2)	38 (27.7)	5 (3.6)	2 (1.5)
Antibiotic treatment not completed	87 (63.5)	43 (31.4)	6 (4.4)	1 (0.7)

To understand the relationship between the duration of practice and the knowledge about antibiotic resistance and its effect on public health, we used the Pearson correlation measure. Correlation illustrates the direction and strength of a relationship between two variables. For example, the relationship between the duration of practice and the knowledge about antibiotic resistance was determined; there is a statistically significant difference in knowledge about antibiotic resistance related to the variable level of training as the *p-* value was 0.04 that is less than 0.05. We use the post hoc tests (Tukey's) to specify groups that differed to eliminate any ambiguity. It was noticed that the knowledge about antibiotic resistance asserts that the senior specialist/consultant is more knowledgeable. We have also found that there is no statistically significant difference in knowledge on antibiotic resistance related to the variable kind of hospitals *as the p* -value is 0.56 that is higher than 0.05.

## Discussion

Even though antibiotics have a great advantage in reducing the incidence and spread of infectious diseases worldwide, they are frequently misused, leading to the spread of multiple resistant bacterial strains. As a result, the antibiotics became ineffective and infections persist in the body, increasing the risk of spreading to other people and leading to longer hospital stays higher medical costs, and increased mortality. Undoubtedly, a better understanding of the importance of antibiotic resistance and its healthcare drawbacks can improve physicians' practices in the field of antibiotic prescriptions, and thus alleviate the spread of bacterial-resistant strains. 16

Our study was conducted in Southern West-Bank Hospitals (Hebron and Bethlehem governmental and nongovernmental hospitals) from a resource-limited setting. Results showed a variation in the knowledge of antibiotic resistance among physicians concerning the cause and the significance of the problem. Our results showed that the knowledge about antibiotic resistance asserts that senior specialist/consultant is more knowledgeable with a high mean of 1.45 followed by general physicians with a mean of 1.35, then senior specialists (1.3) and medical officers/ residents (1.26). These findings are similar to those from another study carried out in a Ghanaian teaching hospital, which showed that senior physicians are more knowledgeable than junior physicians regarding the issue of antibiotic resistance. 15

The majority of questioned physicians perceived antibiotic resistance as a global problem, while half of them considered it a problem in their country and hospital. Approximately 66% perceived it as a problem in their departments. This matches the findings from other studies where respondents agreed that antibiotic resistance is an important problem but less in their departments compared to national and global settings. 6 7 14 17 While our finding differs from the results of a study that investigated Physicians' Perceptions, Beliefs, Attitudes, and Knowledge Concerning Antimicrobial Resistance carried out in a Brazilian Teaching Hospital, where most respondents considered antibiotic resistance to be a problem across all levels. 18 Perceiving antibiotic resistance as a minor problem in the department where the physician is working can weaken the attention paid by them while prescribing antibiotics, which can potentiate the problem.

The causes of antibiotic resistance in our research study were identified and self-medication was the most perceived factor by the physicians causing antibiotic resistance, then the uncompleted antibiotic therapy and overuse of antibiotics in the population, which match the findings of a survey carried out in the Democratic Republic of Congo to investigate knowledge, attitude, and practice among medical doctors and students. 6 However, the overuse of antibiotics in animals was the least perceived as an important cause, similar to the Ghanaian results. 15

We found a weak positive correlation between the duration of practice and knowledge about antibiotic resistance *p* = 0.003). This can be explained by the fact that the lower the years of practice, the closer the physician is to the years of education, and thus the greater amount of information he/she will remember. Moreover, results from a study carried out in Malaysia concluded that a longer duration of practice does not necessarily ensure good knowledge of antibiotic prescribing and antibiotic resistance. 19 In addition, there was a statistically significant difference in knowledge about antimicrobial resistance among different levels of training, with the senior specialist/consultant being the most knowledgeable among the four levels. Similarly, the Ghanaian study showed a difference in knowledge between senior and junior doctors. 15 Our findings can be attributed to the fact that the more the training level the physician achieved, the more cases he/she will deal with, and therefore the more knowledge he or will get. On the contrary, another survey of physicians in France and Scotland found no association between the level of training and knowledge of antibiotic resistance. 14

No difference was found between governmental and private and nongovernmental hospital doctors in their knowledge ( *t* (135)= 0.574, *p* = 0.56). To improve antibiotic use and control antibiotic resistance in the hospital, there is a need to increase education on antibiotic resistance among physicians with an emphasis on junior physicians. Results from our study show the importance of increasing activities aiming to raise physicians' awareness regarding antimicrobial resistance and its major consequences on public health. This can be achieved by encouraging them to attain continuing education/workshops related to antibiotic resistance as only 37% of our respondents have ever attended workshops related to that topic. It is also important to focus on junior residents since they showed inadequate knowledge of antibiotic resistance and to incorporate this issue into the clinical education programs of medical schools. Identification of concerns regarding inappropriate antimicrobial prescribing will enable specific initiatives and approaches to improve future antimicrobial use and reduce antimicrobial resistance in Palestine.

There were several limitations in our study, we are not sure if respondents verified their answers with each other, or if they commissioned others to fill out the questionnaire because, like other self-administered questionnaires, our survey was completed by physicians without supervision to ensure privacy, and to allow physicians to fill it within their free time. Moreover, there is a chance that respondents have been biased in their answers to support the hospital they work at, and gave more socially acceptable answers. As a result, and to influence and guide future interventions, more studies related to antibiotic resistance need to be conducted in this area to prove the reliability of the survey in the West Bank.

## Conclusion

Antibiotic resistance is one of the biggest threats to global health. This study has provided information about prescribing attitudes and practices of physicians working in both governmental and nongovernmental hospitals in South Palestine. Knowledge regarding antibiotic resistance was good among those physicians with junior physicians being the least knowledgeable. Thus, attention has to be focused on conducting lectures and workshops to enrich physicians' knowledge about antibiotic resistance, and as knowledge is the guide of attitudes and practices, this enrichment can lead the community into a better health condition with fewer bacterial-resistant strains threatening our survival. However, for the containment of antimicrobial resistance, it needs different aspects, such as publishing information about antimicrobial resistance rates in Palestine, the development and use of guidelines for antibiotics, and emphasizing the importance of having regular educational activities regarding the appropriate use of antimicrobials.
